# Methyl 7-chloro-2-ethyl­sulfanyl-6-fluoro-4-oxo-4*H*-thio­chromene-3-carboxyl­ate

**DOI:** 10.1107/S1600536810002667

**Published:** 2010-02-27

**Authors:** Yang Li, Tao Xiao, Dong-liang Liu, Guang-yan Yu

**Affiliations:** a Department of Applied Chemistry, College of Science, Nanjing University of Technology, Nanjing 210009, People’s Republic of China

## Abstract

In the title compound, C_13_H_10_ClFO_3_S_2_, the two-ring system is essentially planar, the mean plane of the benzene ring being inclined at 6.0 (2)° to the plane of the remaining four atoms. The ethyl­sulfanyl group is almost coplanar with the two rings [dihedral angle = 6.4 (2)°], while the carboxyl­ate group is almost perpendicular to it [dihedral angle = 72.4 (2)°]. In the crystal structure, inter­molecular C—H⋯O and C—H⋯F hydrogen bonds link the mol­ecules in a stacked arrangement along the *a* axis.

## Related literature

For related compounds containing a 4*H*-thio­chromen-4-one fragment, see: Adams *et al.* (1991 ▶); Nakazumi *et al.* (1992 ▶); Weiss *et al.* (2008 ▶). For bond-length data, see: Allen *et al.* (1987 ▶).
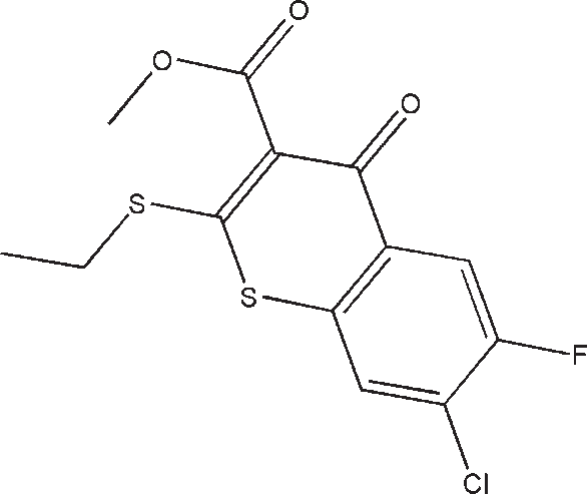


## Experimental

### 

#### Crystal data

C_13_H_10_ClFO_3_S_2_
*M* *_r_* = 332.78
Triclinic, 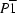

*a* = 7.6740 (15) Å
*b* = 9.3880 (19) Å
*c* = 10.368 (2) Å
α = 85.18 (3)°
β = 80.93 (3)°
γ = 71.24 (3)°
*V* = 698.0 (2) Å^3^
*Z* = 2
Mo *K*α radiation
μ = 0.59 mm^−1^
*T* = 293 K
0.20 × 0.10 × 0.10 mm

#### Data collection

Enraf–Nonius CAD-4 diffractometer
Absorption correction: ψ scan (North *et al.*, 1968 ▶) *T*_min_ = 0.892, *T*_max_ = 0.944
2735 measured reflections
2531 independent reflections
1908 reflections with *I* > 2σ(*I*)
*R*_int_ = 0.016
3 standard reflections every 200 reflections  intensity decay: 1%

#### Refinement

*R*[*F* ^2^ > 2σ(*F* ^2^)] = 0.045
*wR*(*F* ^2^) = 0.138
*S* = 1.00
2531 reflections
181 parameters
H-atom parameters constrained
Δρ_max_ = 0.32 e Å^−3^
Δρ_min_ = −0.31 e Å^−3^

### 

Data collection: *CAD-4 EXPRESS* (Enraf–Nonius, 1994 ▶); cell refinement: *CAD-4 EXPRESS*; data reduction: *XCAD4* (Harms & Wocadlo, 1995 ▶); program(s) used to solve structure: *SHELXS97* (Sheldrick, 2008 ▶); program(s) used to refine structure: *SHELXL97* (Sheldrick, 2008 ▶); molecular graphics: *PLATON* (Spek, 2009 ▶); software used to prepare material for publication: *SHELXTL* (Sheldrick, 2008 ▶).

## Supplementary Material

Crystal structure: contains datablocks I, global. DOI: 10.1107/S1600536810002667/zq2029sup1.cif

Structure factors: contains datablocks I. DOI: 10.1107/S1600536810002667/zq2029Isup2.hkl

Additional supplementary materials:  crystallographic information; 3D view; checkCIF report

## Figures and Tables

**Table 1:** Hydrogen-bond geometry (Å, °)

*D*—H⋯*A*	*D*—H	H⋯*A*	*D*⋯*A*	*D*—H⋯*A*
C2—H2*A*⋯O1^i^	0.93	2.60	3.292 (4)	132
C13—H13*C*⋯F^ii^	0.96	2.52	3.144 (5)	123

Symmetry codes: (i) 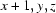; (ii) 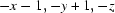.

**Fig. 1.**
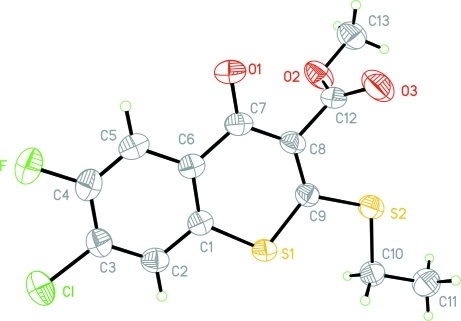
The molecular structure of (I), with the atom-numbering scheme. Displacement ellipsoids are drawn at the 50% probability level.

**Fig. 2.**
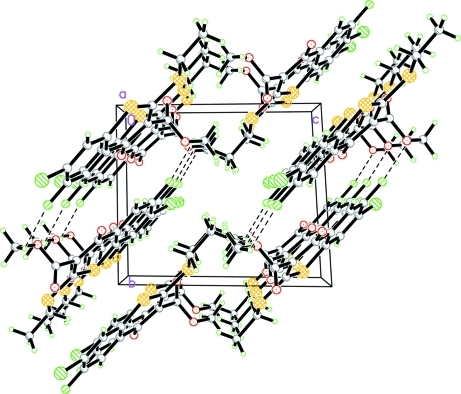
A packing diagram of (I). Hydrogen bonds are shown as dashed lines.

## supplementary crystallographic information

### Comment

The title compound, methyl 7-chloro-2-(ethylsulfanyl)-6-fluoro-4-oxo-4*H*- thiochromene-3-carboxylate (I), is a new molecule which has a potential use as antifungal. We herein report its crystal structure. The molecular structure of (I) is shown in Fig. 1, and selected geometric parameters are given in Table 1. The bond lengths and angles (Table 1) are within normal ranges (Allen *et al.*, 1987). The molecule is essentially planar, the atoms C7, C8, C9 and S1 form a plane inclined at 6.0 (2)° with the mean plane of the phenyl ring. The ethylsulfanyl group is almost coplanar with the two rings while the carboxylate group is almost perpendicular. In the crystal structure, intermolecular C—H···O and C—H···F hydrogen bonds (Table 2) link the molecules in a stacked arrangement along the *a* axis (Fig. 2).

### Experimental

CS_2_ (1.0 g, 13.1 mmol) was dropwise added to a solution of methyl 3-(4-chloro-3-fluorophenyl)-3-oxopropanoate (4 g, 17.3 mmol) in DMSO (20 ml) containing KOH (1 g, 17.8 mmol). The yellow solution was stirred for about 2 h at room temperature. Then bromoethane (1.9 g, 17.3 mmol) was dropwise added to the intermediate. After 3 h, the solution was poured into water (50 ml). The crystalline product was isolated by filtration, washed with water (300 ml). The crystals were obtained by dissolving (I) in acetone (20 ml) and slow evaporation of the solvent at room temperature for about 7 d.

### Refinement

H atoms were positioned geometrically, with O—H = 0.82 and C—H = 0.93 Å for aromatic H, and constrained to ride on their parent atoms, with *U*_iso_(H) = *xU*_eq_(C/O), where *x* = 1.2 for aromatic H and *x* = 1.5 for other H.

### Crystal data

**Table:**

C_13_H_10_ClFO_3_S_2_	*Z* = 2
*M**_r_* = 332.78	*F*(000) = 340
Triclinic, *P*1	*D*_x_ = 1.583 Mg m^−^^3^
Hall symbol: -P 1	Melting point: 421 K
*a* = 7.6740 (15) Å	Mo *K*α radiation, λ = 0.71073 Å
*b* = 9.3880 (19) Å	Cell parameters from 25 reflections
*c* = 10.368 (2) Å	θ = 10–13°
α = 85.18 (3)°	µ = 0.59 mm^−^^1^
β = 80.93 (3)°	*T* = 293 K
γ = 71.24 (3)°	Block, colourless
*V* = 698.0 (2) Å^3^	0.20 × 0.10 × 0.10 mm

### Data collection

**Table:**

Enraf–Nonius CAD-4 diffractometer	1908 reflections with *I* > 2σ(*I*)
Radiation source: fine-focus sealed tube	*R*_int_ = 0.016
graphite	θ_max_ = 25.3°, θ_min_ = 2.0°
ω/2θ scans	*h* = 0→9
Absorption correction: ψ scan (North *et al.*, 1968)	*k* = −10→11
*T*_min_ = 0.892, *T*_max_ = 0.944	*l* = −12→12
2735 measured reflections	3 standard reflections every 200 reflections
2531 independent reflections	intensity decay: 1%

### Refinement

**Table:**

Refinement on *F*^2^	Primary atom site location: structure-invariant direct methods
Least-squares matrix: full	Secondary atom site location: difference Fourier map
*R*[*F*^2^ > 2σ(*F*^2^)] = 0.045	Hydrogen site location: inferred from neighbouring sites
*wR*(*F*^2^) = 0.138	H-atom parameters constrained
*S* = 1.00	*w* = 1/[σ^2^(*F*_o_^2^) + (0.085*P*)^2^] where *P* = (*F*_o_^2^ + 2*F*_c_^2^)/3
2531 reflections	(Δ/σ)_max_ < 0.001
181 parameters	Δρ_max_ = 0.32 e Å^−^^3^
0 restraints	Δρ_min_ = −0.31 e Å^−^^3^

### Special details

**Table:**

Geometry. All e.s.d.'s (except the e.s.d. in the dihedral angle between two l.s. planes) are estimated using the full covariance matrix. The cell e.s.d.'s are taken into account individually in the estimation of e.s.d.'s in distances, angles and torsion angles; correlations between e.s.d.'s in cell parameters are only used when they are defined by crystal symmetry. An approximate (isotropic) treatment of cell e.s.d.'s is used for estimating e.s.d.'s involving l.s. planes.
Refinement. Refinement of *F*^2^ against ALL reflections. The weighted *R*-factor *wR* and goodness of fit *S* are based on *F*^2^, conventional *R*-factors *R* are based on *F*, with *F* set to zero for negative *F*^2^. The threshold expression of *F*^2^ > σ(*F*^2^) is used only for calculating *R*-factors(gt) *etc*. and is not relevant to the choice of reflections for refinement. *R*-factors based on *F*^2^ are statistically about twice as large as those based on *F*, and *R*- factors based on ALL data will be even larger.

### Fractional atomic coordinates and isotropic or equivalent isotropic displacement parameters (Å^2^)

**Table:**

	*x*	*y*	*z*	*U*_iso_*/*U*_eq_	
Cl	0.32919 (13)	0.43804 (11)	−0.25035 (9)	0.0614 (3)	
F	−0.0661 (3)	0.5608 (2)	−0.2592 (2)	0.0679 (6)	
S1	0.13971 (10)	0.07054 (9)	0.12622 (8)	0.0409 (3)	
S2	−0.04493 (12)	−0.08554 (10)	0.33572 (9)	0.0515 (3)	
O1	−0.4369 (3)	0.3010 (3)	0.0513 (2)	0.0541 (6)	
O2	−0.4884 (3)	0.2281 (3)	0.3453 (2)	0.0572 (7)	
O3	−0.4571 (4)	0.0067 (3)	0.2676 (3)	0.0738 (8)	
C1	0.0642 (4)	0.2176 (3)	0.0132 (3)	0.0353 (7)	
C2	0.2053 (4)	0.2628 (3)	−0.0640 (3)	0.0403 (7)	
H2A	0.3289	0.2153	−0.0531	0.048*	
C3	0.1606 (4)	0.3769 (3)	−0.1554 (3)	0.0409 (7)	
C4	−0.0260 (5)	0.4468 (4)	−0.1690 (3)	0.0460 (8)	
C5	−0.1641 (4)	0.4052 (3)	−0.0954 (3)	0.0433 (8)	
H5A	−0.2871	0.4546	−0.1070	0.052*	
C6	−0.1220 (4)	0.2881 (3)	−0.0019 (3)	0.0357 (7)	
C7	−0.2770 (4)	0.2453 (3)	0.0767 (3)	0.0394 (7)	
C8	−0.2374 (4)	0.1375 (3)	0.1842 (3)	0.0364 (7)	
C9	−0.0653 (4)	0.0524 (3)	0.2103 (3)	0.0359 (7)	
C10	0.1994 (4)	−0.1669 (4)	0.3475 (3)	0.0478 (8)	
H10A	0.2680	−0.2038	0.2638	0.057*	
H10B	0.2482	−0.0928	0.3750	0.057*	
C11	0.2151 (5)	−0.2962 (4)	0.4489 (4)	0.0616 (10)	
H11A	0.3432	−0.3439	0.4593	0.092*	
H11B	0.1659	−0.3683	0.4203	0.092*	
H11C	0.1459	−0.2578	0.5310	0.092*	
C12	−0.4059 (4)	0.1142 (4)	0.2676 (3)	0.0435 (8)	
C13	−0.6523 (5)	0.2191 (5)	0.4322 (4)	0.0650 (11)	
H13A	−0.7010	0.3066	0.4843	0.097*	
H13B	−0.6200	0.1305	0.4882	0.097*	
H13C	−0.7445	0.2141	0.3815	0.097*	

### Atomic displacement parameters (Å^2^)

**Table:**

	*U*^11^	*U*^22^	*U*^33^	*U*^12^	*U*^13^	*U*^23^
Cl	0.0607 (6)	0.0691 (6)	0.0557 (6)	−0.0309 (5)	0.0054 (4)	0.0070 (4)
F	0.0703 (15)	0.0607 (13)	0.0659 (13)	−0.0157 (11)	−0.0153 (11)	0.0271 (11)
S1	0.0278 (4)	0.0440 (5)	0.0476 (5)	−0.0082 (3)	−0.0062 (3)	0.0060 (3)
S2	0.0359 (5)	0.0571 (6)	0.0557 (5)	−0.0118 (4)	−0.0047 (4)	0.0164 (4)
O1	0.0323 (12)	0.0591 (15)	0.0699 (16)	−0.0121 (11)	−0.0163 (11)	0.0109 (12)
O2	0.0491 (14)	0.0657 (16)	0.0583 (15)	−0.0270 (12)	0.0139 (12)	−0.0153 (12)
O3	0.0476 (15)	0.0571 (16)	0.119 (2)	−0.0274 (13)	0.0101 (15)	−0.0153 (16)
C1	0.0362 (16)	0.0339 (16)	0.0375 (16)	−0.0114 (13)	−0.0077 (13)	−0.0044 (13)
C2	0.0356 (16)	0.0406 (17)	0.0454 (18)	−0.0115 (14)	−0.0061 (14)	−0.0058 (14)
C3	0.0462 (18)	0.0416 (17)	0.0374 (16)	−0.0179 (15)	−0.0024 (14)	−0.0040 (14)
C4	0.057 (2)	0.0418 (18)	0.0398 (18)	−0.0144 (16)	−0.0116 (16)	0.0019 (14)
C5	0.0384 (17)	0.0396 (18)	0.0499 (19)	−0.0059 (14)	−0.0125 (15)	−0.0032 (15)
C6	0.0349 (16)	0.0340 (15)	0.0384 (16)	−0.0088 (13)	−0.0075 (13)	−0.0052 (12)
C7	0.0306 (16)	0.0392 (17)	0.0467 (18)	−0.0071 (13)	−0.0068 (14)	−0.0048 (14)
C8	0.0293 (15)	0.0395 (16)	0.0429 (17)	−0.0133 (13)	−0.0051 (13)	−0.0050 (13)
C9	0.0346 (16)	0.0373 (16)	0.0369 (16)	−0.0127 (13)	−0.0044 (13)	−0.0025 (13)
C10	0.0395 (18)	0.050 (2)	0.054 (2)	−0.0131 (15)	−0.0134 (15)	0.0088 (16)
C11	0.054 (2)	0.063 (2)	0.065 (2)	−0.0148 (19)	−0.0194 (19)	0.0168 (19)
C12	0.0283 (16)	0.0442 (18)	0.058 (2)	−0.0108 (14)	−0.0100 (14)	0.0046 (16)
C13	0.049 (2)	0.079 (3)	0.059 (2)	−0.019 (2)	0.0107 (18)	0.002 (2)

### Geometric parameters (Å, °)

**Table:**

Cl—C3	1.719 (3)	C5—C6	1.394 (4)
F—C4	1.352 (4)	C5—H5A	0.9300
S1—C9	1.726 (3)	C6—C7	1.480 (4)
S1—C1	1.744 (3)	C7—C8	1.441 (4)
S2—C9	1.744 (3)	C8—C9	1.362 (4)
S2—C10	1.802 (3)	C8—C12	1.505 (4)
O1—C7	1.231 (4)	C10—C11	1.524 (4)
O2—C12	1.320 (4)	C10—H10A	0.9700
O2—C13	1.448 (4)	C10—H10B	0.9700
O3—C12	1.195 (4)	C11—H11A	0.9600
C1—C6	1.396 (4)	C11—H11B	0.9600
C1—C2	1.400 (4)	C11—H11C	0.9600
C2—C3	1.363 (4)	C13—H13A	0.9600
C2—H2A	0.9300	C13—H13B	0.9600
C3—C4	1.394 (5)	C13—H13C	0.9600
C4—C5	1.349 (5)		
			
C9—S1—C1	103.12 (15)	C7—C8—C12	114.8 (3)
C9—S2—C10	106.87 (15)	C8—C9—S1	124.2 (2)
C12—O2—C13	116.1 (3)	C8—C9—S2	119.3 (2)
C6—C1—C2	120.8 (3)	S1—C9—S2	116.45 (17)
C6—C1—S1	124.0 (2)	C11—C10—S2	105.9 (2)
C2—C1—S1	115.1 (2)	C11—C10—H10A	110.6
C3—C2—C1	119.7 (3)	S2—C10—H10A	110.6
C3—C2—H2A	120.2	C11—C10—H10B	110.6
C1—C2—H2A	120.2	S2—C10—H10B	110.6
C2—C3—C4	118.9 (3)	H10A—C10—H10B	108.7
C2—C3—Cl	121.1 (3)	C10—C11—H11A	109.5
C4—C3—Cl	119.9 (3)	C10—C11—H11B	109.5
C5—C4—F	120.0 (3)	H11A—C11—H11B	109.5
C5—C4—C3	122.4 (3)	C10—C11—H11C	109.5
F—C4—C3	117.5 (3)	H11A—C11—H11C	109.5
C4—C5—C6	119.8 (3)	H11B—C11—H11C	109.5
C4—C5—H5A	120.1	O3—C12—O2	123.8 (3)
C6—C5—H5A	120.1	O3—C12—C8	125.7 (3)
C5—C6—C1	118.4 (3)	O2—C12—C8	110.4 (3)
C5—C6—C7	118.3 (3)	O2—C13—H13A	109.5
C1—C6—C7	123.3 (3)	O2—C13—H13B	109.5
O1—C7—C8	120.6 (3)	H13A—C13—H13B	109.5
O1—C7—C6	120.7 (3)	O2—C13—H13C	109.5
C8—C7—C6	118.7 (3)	H13A—C13—H13C	109.5
C9—C8—C7	125.9 (3)	H13B—C13—H13C	109.5
C9—C8—C12	119.1 (3)		
			
C9—S1—C1—C6	−3.7 (3)	C1—C6—C7—C8	7.3 (4)
C9—S1—C1—C2	176.1 (2)	O1—C7—C8—C9	169.8 (3)
C6—C1—C2—C3	−0.2 (4)	C6—C7—C8—C9	−10.5 (5)
S1—C1—C2—C3	180.0 (2)	O1—C7—C8—C12	−6.3 (4)
C1—C2—C3—C4	0.5 (4)	C6—C7—C8—C12	173.4 (3)
C1—C2—C3—Cl	178.9 (2)	C7—C8—C9—S1	6.0 (5)
C2—C3—C4—C5	−0.3 (5)	C12—C8—C9—S1	−178.0 (2)
Cl—C3—C4—C5	−178.8 (3)	C7—C8—C9—S2	−173.7 (2)
C2—C3—C4—F	179.2 (3)	C12—C8—C9—S2	2.3 (4)
Cl—C3—C4—F	0.7 (4)	C1—S1—C9—C8	1.0 (3)
F—C4—C5—C6	−179.6 (3)	C1—S1—C9—S2	−179.22 (16)
C3—C4—C5—C6	−0.2 (5)	C10—S2—C9—C8	−176.9 (2)
C4—C5—C6—C1	0.5 (5)	C10—S2—C9—S1	3.3 (2)
C4—C5—C6—C7	−179.8 (3)	C9—S2—C10—C11	−175.0 (2)
C2—C1—C6—C5	−0.3 (4)	C13—O2—C12—O3	−1.3 (5)
S1—C1—C6—C5	179.6 (2)	C13—O2—C12—C8	−179.4 (3)
C2—C1—C6—C7	180.0 (3)	C9—C8—C12—O3	−70.7 (5)
S1—C1—C6—C7	−0.1 (4)	C7—C8—C12—O3	105.7 (4)
C5—C6—C7—O1	7.3 (4)	C9—C8—C12—O2	107.4 (3)
C1—C6—C7—O1	−173.0 (3)	C7—C8—C12—O2	−76.2 (3)
C5—C6—C7—C8	−172.4 (3)		

### Hydrogen-bond geometry (Å, °)

**Table:**

*D*—H···*A*	*D*—H	H···*A*	*D*···*A*	*D*—H···*A*
C2—H2A···O1^i^	0.93	2.60	3.292 (4)	132
C13—H13C···F^ii^	0.96	2.52	3.144 (5)	123

Symmetry codes: (i) *x*+1, *y*, *z*; (ii) −*x*−1, −*y*+1, −*z*.
